# Analysis of putative heme ligands in the System I bacterial cytochrome *c* biogenesis heme transporter, CcmCD

**DOI:** 10.1128/spectrum.03266-25

**Published:** 2026-04-07

**Authors:** Alicia N. Kreiman, Susan C. Carroll, Nikita P. Varde, Sarah E. Garner, Donna R. Price, Molly C. Sutherland

**Affiliations:** 1 Department of Biological Sciences, University of Delaware 5972, Newark, Delaware, USA; University of Maryland Baltimore County, Baltimore, Maryland, USA

**Keywords:** heme, heme transporter, heme trafficking, cytochrome *c*, cytochrome *c* biogenesis, axial ligand

## Abstract

Heme is a key co-factor for biologically important proteins that are required for critical cellular functions such as oxygen transport and energy production. Yet heme is highly cytotoxic and is proposed to require specific transport pathways to move within cells. Despite its importance, mechanisms for heme transport remain largely uncharacterized due to heme’s cytotoxicity and the transient nature of transport. In recent years, bacterial cytochrome *c* biogenesis pathways, System I and II, have emerged as model systems to probe heme-protein interactions and heme transport across bacterial membranes. In System II, CcsBA is proposed to be a heme transporter based on the identification of two discrete heme interaction domains, one located near the cytoplasm and the other located in the periplasm. These domains each use two histidine residues to ligand the heme. In System I, CcmCD was recently designated as a heme transporter, and the path of heme transport was biochemically defined. Based on the cryo-EM structure of CcmCD, four residues near the cytoplasm could act as axial ligands to heme. Thus, a structure-function analysis of these putative heme ligands was undertaken to determine if CcmCD and CcsBA interact with heme in a similar manner. This analysis revealed that CcmCD does not contain heme axial ligands nor is heme liganding required for CcmCD heme transport. These results provide the first experimental evidence that heme transporters do not interact with heme nor transport heme by a conserved mechanism.

**IMPORTANCE:**

Heme is a critical co-factor for proteins with essential biological functions including gas sensing and electron transport. However, free or unbound heme is highly toxic to cells. To mitigate toxicity, there must be specific pathways to move heme in cells. While heme transport proteins have been identified genetically, their mechanism of transport remains elusive. The bacterial cytochrome *c* biogenesis pathways, Systems I and II, are excellent model systems to investigate heme transport. We compare the System I heme transporter, CcmCD, with the System II heme transporter, CcsBA. Analysis of putative transmembrane localized heme axial ligands in CcmCD determined that heme liganding is not required for heme transport. In contrast, CcsBA requires transmembrane heme axial liganding for function. Thus, the bacterial cytochrome *c* biogenesis heme transporters have different mechanisms, suggesting that additional mechanisms of transmembrane transport may be uncovered via experimental analysis of other heme transporters in prokaryotes and eukaryotes.

## INTRODUCTION

Bacterial cytochrome *c* biogenesis pathways have emerged as a model system to study general mechanisms of heme trafficking, modification, and attachment due to the ability to recombinantly express and affinity purify these proteins with endogenous heme (1–11). Briefly, cytochrome *c* biogenesis requires the covalent attachment of heme to a conserved CXXCH motif on apocytochrome *c* (reviewed in references 12–19). Heme attachment is proposed to confer stability to cytochromes *c* (20, 21). Cytochromes *c* are encoded by nearly all organisms, have highly diverse protein structures, can have from 1 to over 10 CXXCH heme attachment motifs, and function in critical cellular processes such as respiration and photosynthesis. Despite this diversity, all cytochrome *c* biogenesis is accomplished by three main pathways called System I (CcmA-H/I; α, γ Proteobacteria; plant, protozoal mitochondria; Archaea), System II (CcsBA; Gram positive; cyanobacteria; chloroplasts; ε Proteobacteria), and System III (HCCS; eukaryotic mitochondria) (reviewed in references 12–19).

**Fig 1:**
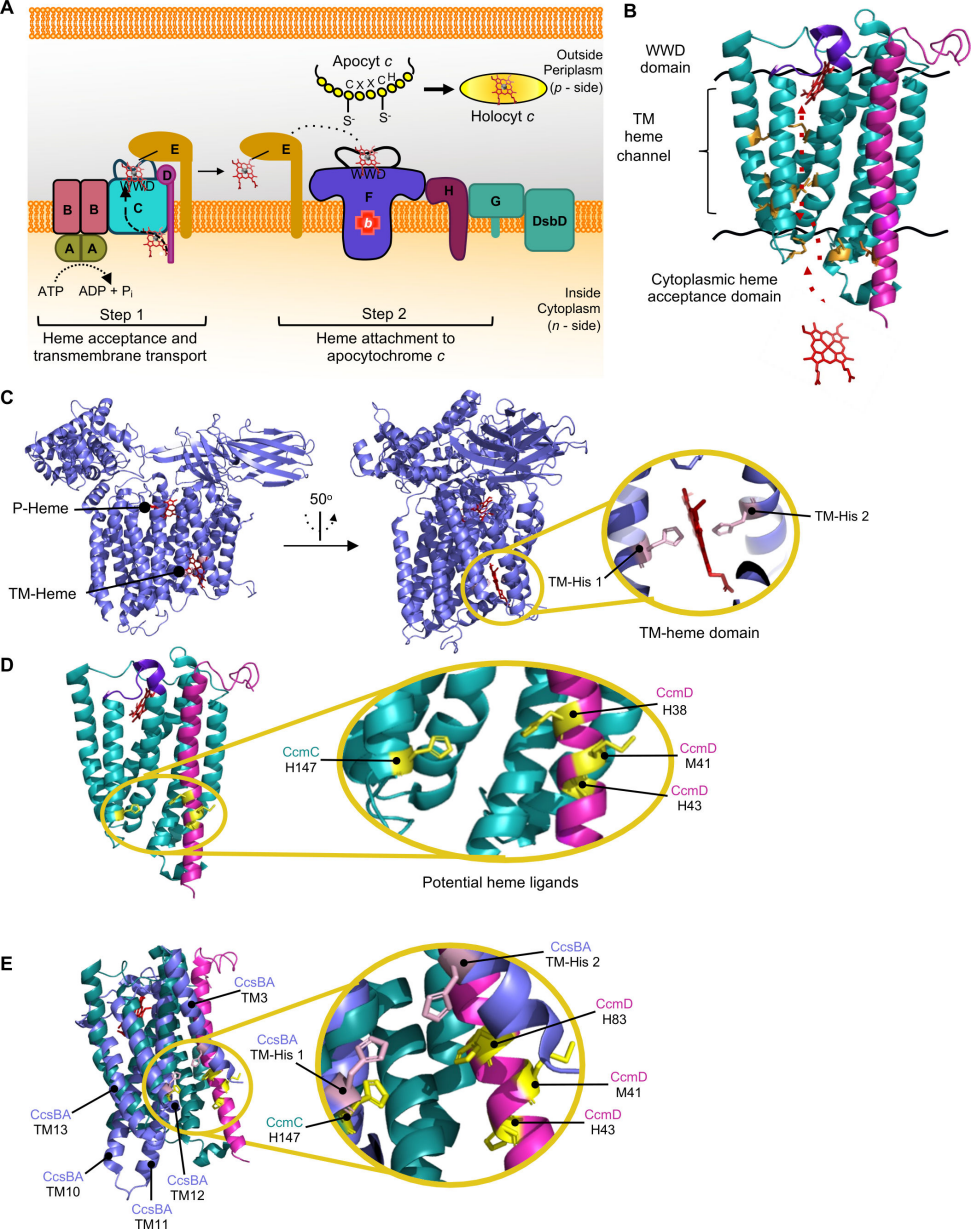
Heme acceptance by the CcmCD heme transporter. (**A**) Proposed model for bacterial cytochrome *c* biogenesis by the System I pathway. Step 1: CcmCD transports heme across the bacterial membrane to the CcmC WWD domain for heme attachment to CcmE. ATP hydrolysis via CcmAB results in release of holoCcmE. Step 2: CcmE transfers heme to the holocytochrome *c* synthase, CcmFH for attachment to apocytochrome *c*. (**B**) Schematic of the experimentally determined path of heme transport by CcmCD—red arrows. CcmC—cyan, CcmD—magenta. Residues shown to interact with heme—gold. Heme—red. PDB: 7F04 (7) was used to generate image in PyMOL (version 3.6.1). (**C**) CcsBA contains two heme interaction domains, the P-heme and TM-heme domains. TM-His 1 and TM-His 2 are axial ligands to heme. PDB: 7S9Y (6) was used to generate the image in PyMOL. (**D**) The potential heme ligands CcmC H147, CcmD H38, M41, and H43 are indicated in yellow with amino acid structures shown. PDB: 7S9Y (6) was used to generate image in PyMOL. (**E**) Structural alignment of CcmCD (PDB: 7S9Y [6]) and CcsBA (PDB: : 7S9Y [6]) aligned at the WWD domain core to show heme axial ligand positioning. For CcsBA TMD 3 and the WWD “core region” TMD 10, 11, 12, 13 are shown, the rest of the protein is hidden. CcmC—cyan, CcmD—magenta, CcmCD putative heme axial ligands—yellow with amino acid structure shown. CcsBA—purple, TM-His axial ligands—pink with amino acid structure shown.

Here, we focus on System I, which is composed of CcmABCDEFGH in *E. coli* and is proposed to function in two main steps (Fig. 1A). In step 1, CcmCD transports heme across the bacterial membrane to the periplasmic CcmC WWD domain (2, 5, 7–9). Heme is attached to CcmE at residue H130 (1, 22–25), and ATP hydrolysis via CcmAB releases holoCcmE (7, 26, 27). In step 2, holoCcmE delivers heme to CcmFH, the holocytochrome *c* synthase, for attachment to cytochrome *c* (23, 28, 29). Recently, CcmCD was determined to be the System I heme transporter (9). The CcmCD heme transport pathway was biochemically defined using the cysteine/heme crosslinking approach. Cysteine/heme crosslinking exploits the natural propensity for cysteine and heme to form a covalent bond (i.e.*,* crosslink) in close proximity (5) and had been previously used to define heme-protein interaction domains (5, 9, 30–32). Cysteine/heme crosslinking was used to covalently trap heme transport intermediates within CcmCD, defining three distinct heme interaction domains: the heme acceptance domain, the enclosed transmembrane heme transport channel (9), and the periplasmic WWD domain (5) (Fig. 1B).

Kreiman et al. hypothesized that after heme reception by CcmCD on the cytoplasmic face of the inner membrane, the hydrophobic nature of the heme channel would drive heme transport to the CcmC WWD domain (9). This proposed mechanism of heme transport by CcmCD is distinct from the model of heme transport proposed for the System II bacterial cytochrome *c* biogenesis pathway. System II is composed of two proteins, CcsBA (also called ResBC) (33–39), which is proposed to be a bi-functional enzyme for heme transport across the membrane and heme attachment to cytochrome *c* (6, 30, 32, 40–42). Genetic, biochemical, and structural evidence has defined two CcsBA heme binding domains: the TM-heme domain consisting of two conserved histidines in the lower transmembrane domain and the P-heme domain consisting of the periplasmic WWD domain and two conserved periplasmic histidines (6, 30, 32, 40, 42) (Fig. 1C). The conserved histidines in these domains function as axial ligands to the heme (6, 10, 30, 40). Heme is proposed to be trafficked from the TM-heme domain to the P-heme domain (6, 15, 40). Thus, current models suggest that mechanisms of heme transport are different in System I and System II. However, analysis of the CcmCD cryo-EM structures (7, 8) presented here suggests that *E. coli* CcmCD may possess heme ligands analogous to the CcsBA TM-heme domain (Fig. 1D and E). Therefore, in this study, we analyze putative transmembrane heme ligands in CcmCD to experimentally determine if heme transport differs between the bacterial cytochrome *c* biogenesis heme transporters CcmCD and CcsBA.

## RESULTS

### Structural analysis reveals putative heme ligands in CcmCD

Analysis of the *E. coli* CcmCD from recently published cryo-EM structures of CcmABCD/E (7, 8) and the biochemically mapped heme channel (9) revealed four potential heme ligands near the CcmCD heme acceptance domain: CcmC H147, CcmD H38, M41, H43 (Fig. 1D). Although histidine is the most common heme axial ligand, amino acids such as methionine, tyrosine, and lysine can also serve as axial ligands (43); therefore, CcmD M41 was included in these studies. While CcmCDs generally have low sequence identity among bacteria that encode System I (9), the structural positioning of these residues was intriguing. This is particularly evident in the structural overlay of the cryo-EM structure of CcmCD with relevant transmembrane domains of CcsBA (TMD 3—contains TM-His1; TMDs 10, 11, 12, 13—form the WWD “core region”) which reveal similar positioning of the putative CcmCD heme ligands with known CcsBA heme ligands TM-His1, TM-His2 (Fig. 1E). The previously characterized WWD “core region” of CcmC and CcsBA, which consists of the four transmembrane domain surrounding the WWD domain encoded by both proteins (30, 31), was used to align the overlay. Here, we test the hypothesis that CcmC H147 and CcmD H38, M41, or H43 function as heme axial ligands and are required for CcmCD heme transport.

### Analysis of putative heme ligands in the GST:CcmCDE background

**Fig 2:**
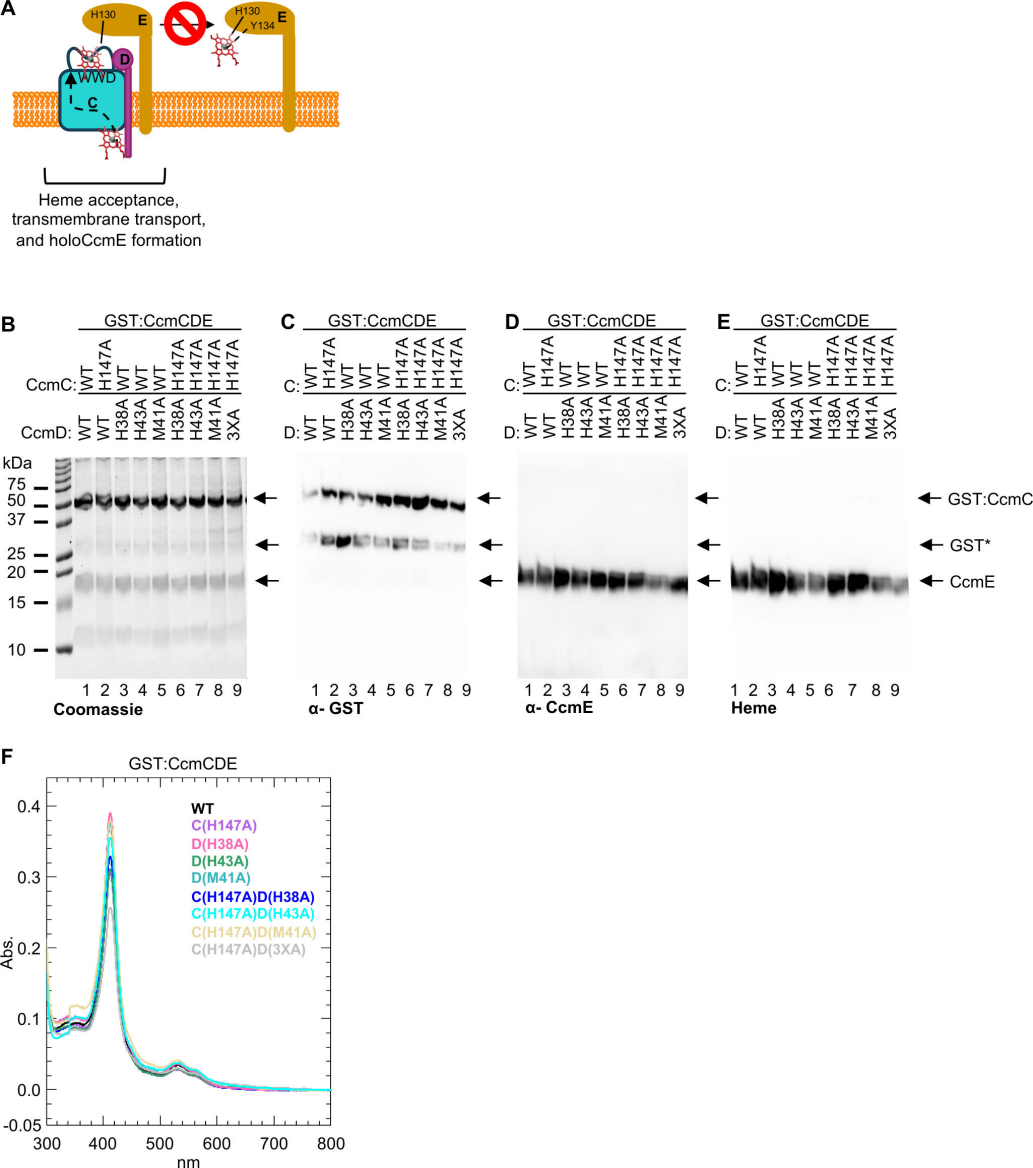
Analysis of putative heme ligands in the CcmCDE genetic background. (**A**) Schematic of CcmCDE function. Heme is transported and attached to CcmE at residue H130. HoloCcmE is not released due to the absence of CcmAB. (**B–E**) Indicated single, double, and triple alanine substitutions were engineered in CcmC and/or CcmD. Five micrograms of affinity purified protein was separated via SDS-PAGE and assessed via (**B**) Coomassie total protein stain, (**C**) α-GST, (**D**) α-CcmE, and (**E**) heme-stain. GST*—proteolyzed GST. Representative of three independent affinity purifications. (**F**) Fifty micrograms of affinity purified protein was used to determine the relative heme co-purification with GST:CcmCD wild type or alanine variants using the Soret peak (~412 nm) absorbance from as purified UV-vis spectra. Representative of three independent affinity purifications.

CcmABCDE comprise Step 1 of System I cytochrome *c* biogenesis where heme is transported across the membrane by the CcmCD heme transporter and attached to CcmE at residue H130. ATP hydrolysis via CcmAB releases holoCcmE (7, 26, 27) (Fig. 1A; Fig. S1). In the absence of CcmAB, heme is still transported across the membrane and attached to CcmE, but holoCcmE is not released (1, 2, 5, 22, 26) (Fig. S1). Thus, the CcmCDE genetic background is ideal to test our hypothesis as heme transport mechanisms are intact and levels of holoCcmE formation can be used as a readout for functional heme transport (Fig. 2A; Fig. S1).

In System II, CcsBA, mutation of the transmembrane localized heme axial ligands, TM-His 1 or TM-His 2, to alanine or glycine results in reduced heme co-purification (<15% of WT [30]) and loss of the ability to attach heme to cytochrome *c*, rendering CcsBA non-functional (6, 30, 32, 40). Similarly, CcmF, the System I holocytochrome *c* synthase, contains transmembrane histidines (TM-His 1 and TM-His 2) that are axial ligands to a membrane localized stable *b*-heme (2, 44, 45). Mutation of the CcmF TM-His 1 and TM-His 2 results in <20% heme co-purification compared to wild type and renders CcmF non-functional for the attachment of heme to apocytochrome *c* (44). Based on CcsBA and CcmF transmembrane heme liganding analysis, it was expected that mutation of putative CcmCD heme ligands would result in a consistent decrease in heme co-purification and a loss of CcmCD function (i.e., heme attachment to CcmE). Thus, to test if a similar mechanism of heme liganding occurs in CcmCD, single amino acid alanine substitutions were engineered in GST:CcmCDE, resulting in CcmC H147A, CcmD H38A, M41A, and H43A.

GST affinity purification of the CcmC/D alanine substitutions revealed that the variants are stable (Fig. 2B, lanes 1–5), contain the GST tag (Fig. 2C, lanes 1–5), co-purify with CcmE (Fig. 2D, lanes 1–5, S2A-F lanes 1–5), and form holoCcmE as indicated by heme attachment to the CcmE polypeptide (Fig. 2E, lanes 1–5, S2G-L lanes 1–5). Note, proteolysis of the n-terminal GST tag occurs in a small population of GST:CcmCDE (Fig. 2B and C designated as GST*). Recall that we hypothesized that if CcmC H147 and CcmD H38, M41, or H43 were heme ligands that CcmCD heme co-purification would decrease and holoCcmE would not be formed. While there was not a complete abrogation of heme co-purification nor holoCcmE formation, quantification was performed by densitometry with normalization to wild type to determine if any decrease was observed (Fig. S2D through F, J through L). Three independent biological replicates did display variation in heme co-purification and holoCcmE formation levels (Fig. S2); however, there was not a clear nor consistent deficiency for any CcmC/D variants, thus such variation represents expected biological variability.

An alternative approach to examine heme co-purification levels was also undertaken. In this approach, the amount of co-purified heme was determined by the absorbance of the Soret (412 nm) region from the as-purified UV-vis spectra using 50 µg of affinity-purified protein (Fig. 2F; Fig. S3). Three independent biological replicates did not reveal a consistent defect in heme co-purification for any CcmC/D variants (Fig. S3). Similarly, holoCcmE formation was also examined via UV-vis spectral analysis with 50 µg of affinity-purified protein. HoloCcmE forms a characteristic split alpha peak upon reduction with sodium dithionite (2) (Fig. S4A, red line). This split alpha peak is retained in the single alanine substitutions (Fig. SB through E), demonstrating that the heme environment, and formation of holoCcmE, is unperturbed by the alanine substitutions. Heme co-purification and formation of holoCcmE indicate that the single alanine variants do not impact heme interaction, heme transport, nor holoCcmE formation via CcmCD.

Positioning of residues in the CcmCD cryo-EM structure suggested that CcmC H147 was one potential heme ligand, and the CcmD H38, M41, or H43 could be the second ligand (Fig. 1D). Thus, double alanine substitutions were constructed to determine if a single ligand was sufficient for protein function, and therefore, the removal of both heme ligands was required to perturb heme transport and holoCcmE formation: CcmC H147A/CcmD H38A, CcmC H147A/CcmD M41A, and CcmC H147A/CcmD H43A. Additionally, alanine substitutions at all potential heme ligands were constructed resulting in a quadruple mutant CcmC H147A/CcmD H38A/M41A/H43A (designated as CcmC H147A/CcmD 3XA). These variants were analyzed as described above for protein stability (Fig. 2B, lanes 6–9), GST tag (Fig. 2C, lanes 6–9), co-purification with CcmE (Fig. 2D, lanes 6–9, S2A-F lanes 6–9), formation of holoCcmE (Fig. 2E, lanes 6–9, S2G-L lanes 6–9, S4F-I), and co-purification with heme (Fig. 2F; Fig. S3). Similar to the single alanine variants described above, the double and quadruple alanine variants displayed variability in heme co-purification (Fig. S3) and holoCcmE formation (Fig. S2G through L and S4F through I), but no consistent defect was observed, thus observed differences across replicates are due to biological variation. This analysis indicates these residues do not act as heme ligands and are not required for heme transport.

### Analysis of putative heme ligands in GST:CcmCDE(H130A) background

**Fig 3:**
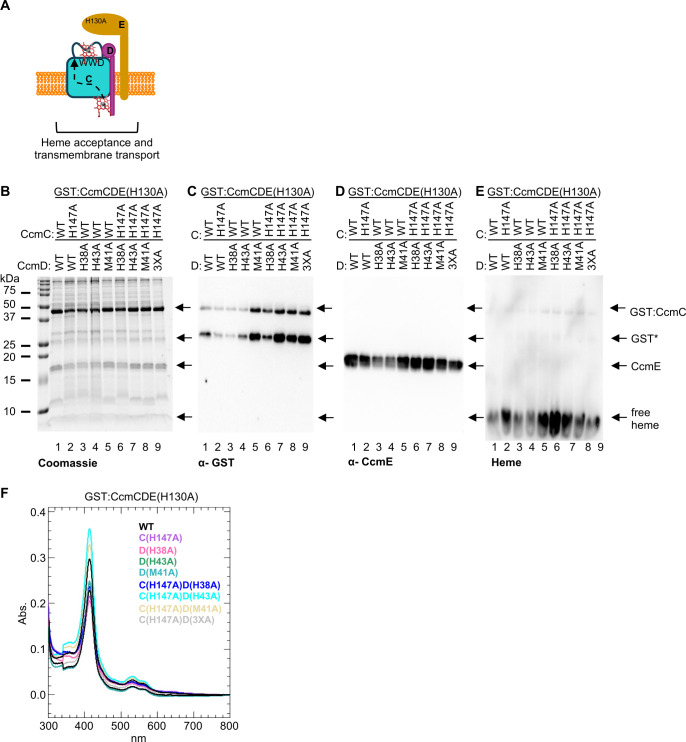
Analysis of putative heme ligands in the CcmCDE(H130A) background. (**A**) Schematic of CcmCDE(H130A) function. Heme is transported to the CcmC WWD domain but not attached to CcmE. (**B–E**) Indicated single, double, and triple alanine substitutions were engineered in CcmC and/or CcmD in the CcmCDE(H130A) genetic background. Five micrograms of affinity purified protein was separated via SDS-PAGE and assessed via (**B**) Coomassie total protein stain, (**C**) α-GST, (**D**) α-CcmE, and (**E**) heme-stain. GST*—proteolyzed GST. Representative of three independent affinity purifications. (**F**) Fifty micrograms of affinity purified protein was used to determine the relative heme co-purification with GST:CcmCDE(H130A) wild type or the alanine variants using the Soret peak height. Representative of three independent affinity purifications.

In System II, it has been proposed that the majority of co-purified heme is retained in the TM-heme domain. Thus, mutating heme ligands, TM-His1 or TM-His2, results in a reduction of heme co-purification and a non-functional CcsBA (6, 30, 40). In contrast, in CcmCDE, it is likely that the formation of the CcmE-heme bond is preferred over retention of heme in CcmCD (5). Therefore, we postulated that in the CcmCDE(H130A) background (Fig. S1 and S3A), increased heme would be retained in CcmCD because holoCcmE cannot be formed. As a result, this genetic background increases the probability that a role for heme liganding via CcmC H147 and CcmD H38, M41, or H43 could be identified. Single, double, and quadruple alanine substitutions were engineered in the GST:CcmCDE(H130A) genetic background and analyzed as described in Fig. 2. Briefly, all alanine variants are stable (Fig. 3B), have the GST tag (Fig. 3C), and co-purified with CcmE (Fig. 3D; Fig. S5A through F). The amount of co-purified CcmE displayed variability across three biological replicates; however, no CcmC/D variant displayed a consistent defect; thus, differences are due to biological variation. The CcmCDE(H130A) alanine variants co-purified with heme at levels similar to wild type CcmCDE(H130A) (Fig. 3E and F; Fig. S5G through L and S6). UV-vis spectral analysis was performed with 50 µg of affinity-purified protein and indicated that the heme environment is not disrupted (Fig. S7). These results provide further evidence that CcmC H147, CcmD H38/M41/H43 do not act as heme ligands.

### Impact of CcmC/D alanine variants on cytochrome *c* biogenesis

**Fig 4:**
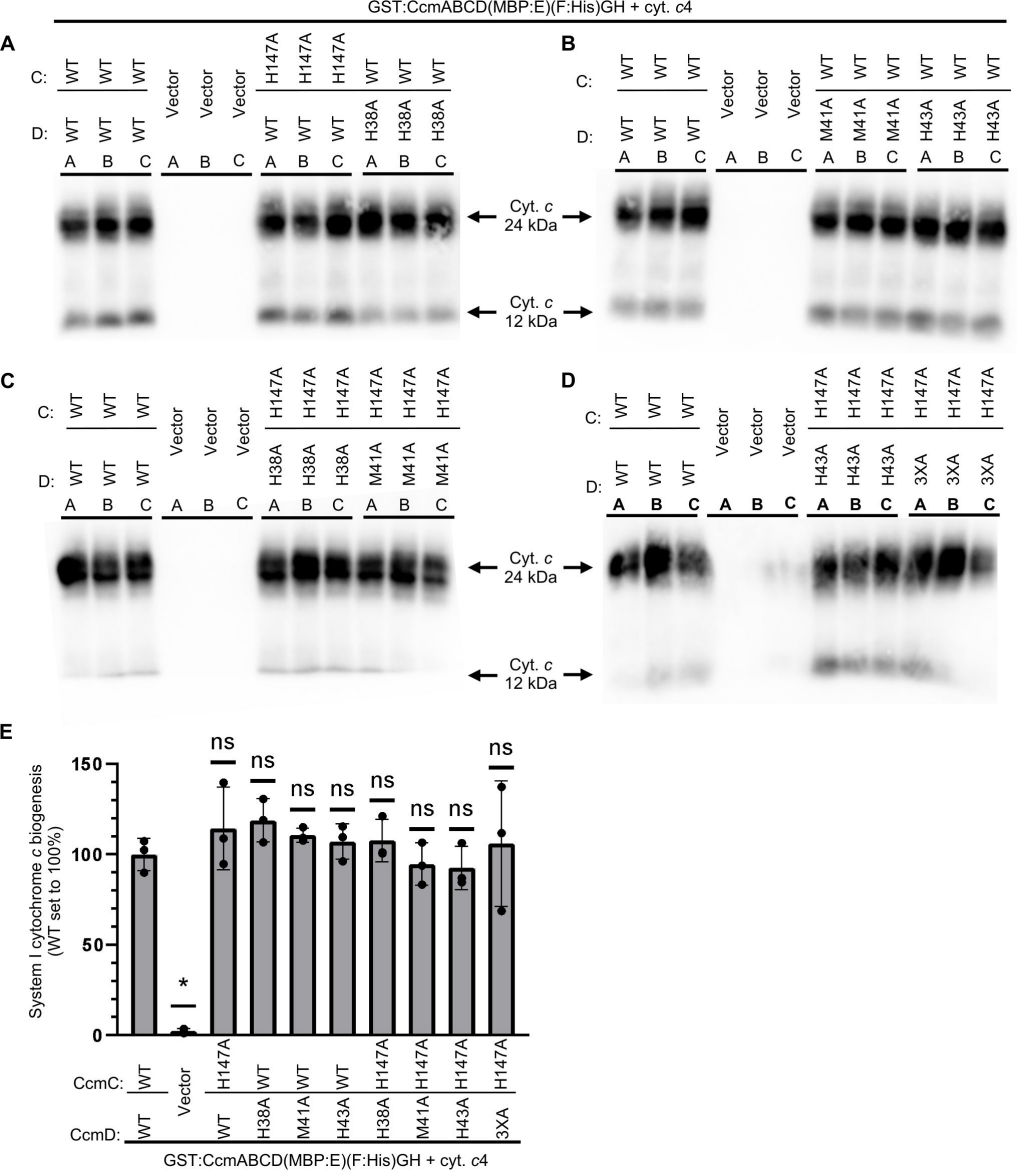
Impact of CcmC/D alanine variants on cytochrome *c* biogenesis. (**A–D**) CcmC/D alanine variants were constructed in the context of the complete System I pathway. Wild-type or alanine variants were co-expressed with cytochrome *c*_4_ in *E. coli* Δ*ccm,* and the amount of cytochrome *c* biogenesis was determined by the separation of total cell lysate via SDS-PAGE and heme stain (24 kDa—full-length holocytochrome *c*; 12 kDa—endogenously proteolyzed). (**E**) Quantification of relative levels of cytochrome *c* biogenesis. Wild type was normalized to 100%. Bars—the average of three technical replicates, error bars—standard deviation from the mean, dots—technical replicate. Representative of three independent biological replicates, each containing three technical replicates. Heme stains were quantified with AzureSpot software (v2.2.167). Statistical analysis was performed with GraphPad Prism (v. 10.6.0) and an unpaired *t*-test. n.s. = not statistically significant, *P < 0.0001.

To determine if CcmC H147, CcmD H38, M41, or H43 is required for cytochrome *c* biogenesis, single, double, and quadruple alanine variants were constructed in the full System I pathway (GST:CcmABCD(MBP:E)F:His(GH)). In order for cytochrome *c* biogenesis to occur, holoCcmE must be properly formed at levels comparable to the wild-type System I pathway (1, 22, 29, 46). Thus, this assay provides an alternative approach to assess the CcmCD variant functions for heme transport and formation of holoCcmE. The complete System I pathway with wild type or CcmC/D alanine variants was co-expressed with cytochrome *c*_4_:His in *E. coli* Δ*ccm*. Cytochrome *c* biogenesis (i.e., heme attachment) was assessed via heme stain (Fig. 4). All of the CcmC/D alanine variants had ~wild-type levels of cytochrome *c* biogenesis (defined as >80% wild-type function). Thus, CcmC H147, CcmD H38, M41, or H43 residues are not required for cytochrome *c* biogenesis.

## DISCUSSION

Here, we tested the hypothesis that the System I heme transporter, CcmCD, requires transmembrane heme liganding for heme transport. This hypothesis was based on the structural positioning of putative heme ligands CcmC H147 and CcmD H38, M41, or H43, which are positioned comparable to the well-characterized System II heme ligands TM-His1 and TM-His 2 (Fig. 1C through E). However, biochemical (Fig. 2 and 3; Fig. S2 through S7) and functional (Fig. 4) analysis revealed that these residues are not required for CcmCD heme interaction, heme transport, holoCcmE formation, nor cytochrome *c* biogenesis. Thus, CcmCD does not require transmembrane heme liganding for heme transport nor for protein function. It remains most likely that once heme is delivered to the CcmCD heme acceptance domain, the hydrophobicity of the heme channel facilitates heme transport to the WWD domain where it can be stably positioned for subsequent attachment to CcmE.

While these results do not support our hypothesis, these biochemical studies provide important insight into the mechanism of heme transport by CcmCD. Although biochemical evidence for System II heme transport still awaits experimental determination, it is clear that heme liganding is critical for System II heme interaction and protein function (6, 10, 30, 32, 40). These studies allow for initial comparison of CcmCD and CcsBA heme transport mechanisms and demonstrate that CcmCD and CcsBA transmembrane heme transport occur via mechanistically distinct pathways. While other heme transporters (e.g., FLVCRs [47–49], HRGs [50, 51], MRPs [52, 53]) and heme exporters (e.g., HrtAB [54], CydDC [55, 56]) have been identified genetically, their mechanisms of heme transport remain largely uncharacterized. Comparison of additional heme transporter mechanisms awaits further experimental data. It is intriguing to consider that multiple mechanisms of heme transport have evolved to ensure this biologically important process is accomplished.

## MATERIALS AND METHODS

### Bacterial growth conditions

*Escherichia coli* strains were grown at 37°C, 200 rpm in Luria-Bertani (LB, Difco) broth with appropriate selective antibiotics (carbenicillin, 50 µg/mL; chloramphenicol, 20 µg/mL) and/or inducing reagents (isopropyl-D 1-thiogalactopyranoside [IPTG; GoldBio], 1.0 or 0.1 mM; L-arabinose [Goldbio], 0.2% [wt/vol]). A list of strains and plasmids is provided in Table S1.

### Construction of alanine variant strains and plasmids

Cloning was performed in *E. coli* NEB-5α using QuikChange II site-directed mutagenesis (Agilent technologies) according to manufacturer’s instructions. Alanine substitutions were verified via DNA sequencing. A list of primers and templates for cloning is provided in Table S1.

### Protein purifications

RK103 *E. coli* Δ*ccm* was used for recombinant protein expression. Note: no exogenous heme was added to cultures or during purification. GST affinity purifications of GST:CcmCDE or GST:CcmCDE(H130A) were performed as previously described (9). Briefly, 1:100 dilution in 1 L LB from a saturated starter culture was grown at 37°C and 200 rpm for 4 h with appropriate antibiotics. Cultures were induced with 1 mM IPTG and grown for an additional ~16–18 h. Cell pellets were harvested by centrifugation and stored at −80°C. Pellets were resuspended in GST buffer (4.3 mM Na_2_HPO_4_, 1.5 mM KH_2_PO_4_, 2.7 mM KCl, 140 mM NaCl, pH7.3) with 1 mM phenylmethanesulfonyl fluoride (PMSF, Sigma-Aldrich, P-470-10) and 1 mg/mL egg white lysozyme (GoldBio, L-040-10). Cell lysis was performed via sonication (Branson SFX250 sonicator), and cell debris was cleared by centrifugation (10,000 rpm for 1 h at 4°C), followed by separation of soluble and membrane fractions via high-speed ultracentrifugation (100,000 *g* for 45 min at 4°C). Membrane pellets were stored at −80°C and then solubilized in GST buffer supplemented with 1% n-dodecyl-β-d-maltopyranoside (DDM, GoldBio, DDM25). Proteins were batch affinity purified with glutathione agarose (Pierce, G-250-5) for 2 h at 4°C. Columns were washed by gravity flow and eluted in 4 mL GST buffer supplemented with 0.02% DDM and 10 mM L-glutathione (Sigma-Aldrich, G4251). Proteins were concentrated using a 30 kDa filter. Bradford Assay (Sigma-Aldrich, PI23200) was used to determine protein concentrations.

### Heme staining, immunoblotting, and quantification

Protein samples were separated via SDS-PAGE (100 V until dye front reached the bottom of the apparatus). For heme stain analysis, SDS-PAGE was transferred to 0.2-micron nitrocellulose membrane using wet transfer (75 min at 85 V). For immunoblot analysis, SDS-PAGE was transferred to a 0.45-micron nitrocellulose membrane via semi-dry transfer (BioRad TurboBlot Standard SD manufacturer protocol [up to 1.0 A; 25 V constant] for 30 min).

For heme stains, 5 µg of affinity-purified protein was analyzed with enhanced chemiluminescent (ECL)-based development and CCD imaging (57, 58). For immunoblots, 5 µg of affinity-purified protein was probed with the indicated antibodies: α-GST (1:30,000, Invitrogen, PA1-982A), α-CcmE (59) (1:90,000). Protein A peroxidase (Millipore Sigma, P8651) was used as a secondary antibody. Imaging was performed with the Azure Sapphire Biomolecular Imager (Azure, SPC11-0239) and quantified with AzureSpot Software (Azure, v.2.2.167). Statistical analysis was performed with GraphPad Prism (v. 10.6.0).

### UV-vis spectroscopy

A UV-1900i was used to collect UV-vis spectra with LabSolutions software (Shimadzu; LabSolutions UV-vis [v1.10]) with 50 µg affinity purified protein. Analysis of relative heme co-purification levels was determined by comparison of the as-purified Soret peak. Reduced spectra were performed by the addition of excess sodium hydrosulfite powder (Sigma, 157-953). UV-vis spectra are representative of three independent protein purifications.

### *In vivo* cytochrome *c* biogenesis assays

The CcmC/D alanine variants were engineered in the context of the full System I pathway (GST:CcmABCD(MBP:E)(F:His)GH) and co-expressed with cytochrome *c*_4_:His in *E. coli* Δ*ccm* as previously described (58, 59). Briefly, 1 mL of saturated overnight cultures was back diluted 1:6 into 5 mL LB with appropriate antibiotics and grown for 3 h (37°C and 200 rpm). 0.1 mM IPTG and 0.2% arabinose were added for protein induction and grown for an additional 3 h (37°C and 200 rpm). Cell pellets were collected by centrifugation (3,700 rpm for 10 min at 4°C) and frozen at −80°C. Cell lysis was performed with Bacterial Protein Extraction Reagent (B-PER, Thermo Scientific, PI78248) per manufacturer’s instructions. Fifty micrograms of total cell lysate was separated by SDS-PAGE. An ECL-based heme stain was used to monitor levels of cytochrome *c* biogenesis (i.e., heme attachment to cytochrome *c*) (57). Three biological replicates, each containing three technical replicates, were performed.

## SUPPLEMENTAL MATERIAL

Supplemental material spectrum.03266-25-s0001.pdf Fig. S1 to S7; Table S1.

ASM does not own the copyrights to Supplemental Material that may be linked to, or accessed through, an article. The authors have granted ASM a non-exclusive, world-wide license to publish the Supplemental Material files. Please contact the corresponding author directly for reuse.
