# Supplementary material for: Analysis of putative heme ligands in the System I bacterial cytochrome c biogenesis heme transporter, CcmCD
Source: Microbiol Spectr. 2026 Apr 7;14(5):e03266-25. doi: 10.1128/spectrum.03266-25 (PMC13141917; doi:10.1128/spectrum.03266-25)
Supplement: Supplemental material — Fig. S1 to S7; Table S1. [file spectrum.03266-25-s0001.pdf]

Supplemental Information

Title: Analysis of putative heme ligands in the System I bacterial cytochrome *c* biogenesis heme transporter, CcmCD

Authors: Alicia N. Kreiman[a], Susan C. Carroll[a], Nikita P. Varde[a], Sarah E. Garner[a], Donna R. Price[a], Molly C. Sutherland[a,#]

[a]Department of Biological Sciences, University of Delaware, Newark, Delaware, United States of America

[#]Corresponding author

Email: msuther@udel.edu

Table of Contents

SUPPLEMENTAL FIGURE 1 3
SUPPLEMENTAL FIGURE 2 4
SUPPLEMENTAL FIGURE 3 6
SUPPLEMENTAL FIGURE 4 7
SUPPLEMENTAL FIGURE 5 8
SUPPLEMENTAL FIGURE 6 10
SUPPLEMENTAL FIGURE 7 11
SUPPLEMENTAL TABLE 1 12
SUPPLEMENTAL REFERENCES 14

Supplemental Figure 1

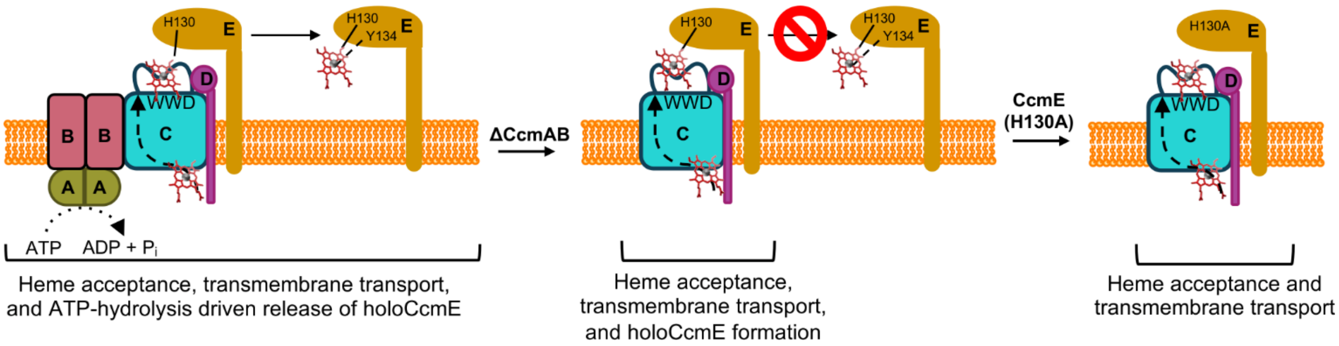

Supplemental Figure 1. CcmABCDE functions in step 1 of System I bacterial cytochrome *c* biogenesis. (A) CcmABCDE form a protein subcomplex that is responsible for heme transport across the bacterial membrane and heme attachment to CcmE at residue H130. HoloCcmE is released from CcmABCD via CcmAB driven ATP hydrolysis. (B) In the absence of CcmAB, CcmCD can transport heme and form holoCcmE. HoloCcmE is not released and remains as protein subcomplex with CcmCD. Thus, CcmAB is required for holoCcmE release, but not for heme transport or attachment. (C) CcmE forms a covalent bond to heme at residue H130. Mutation of H130 to A prevents holoCcmE formation and heme is retained in the CcmCD. Figure modified from Kreiman *et al*. mBio 2025 Fig. S1 (1).

Supplemental Figure 2

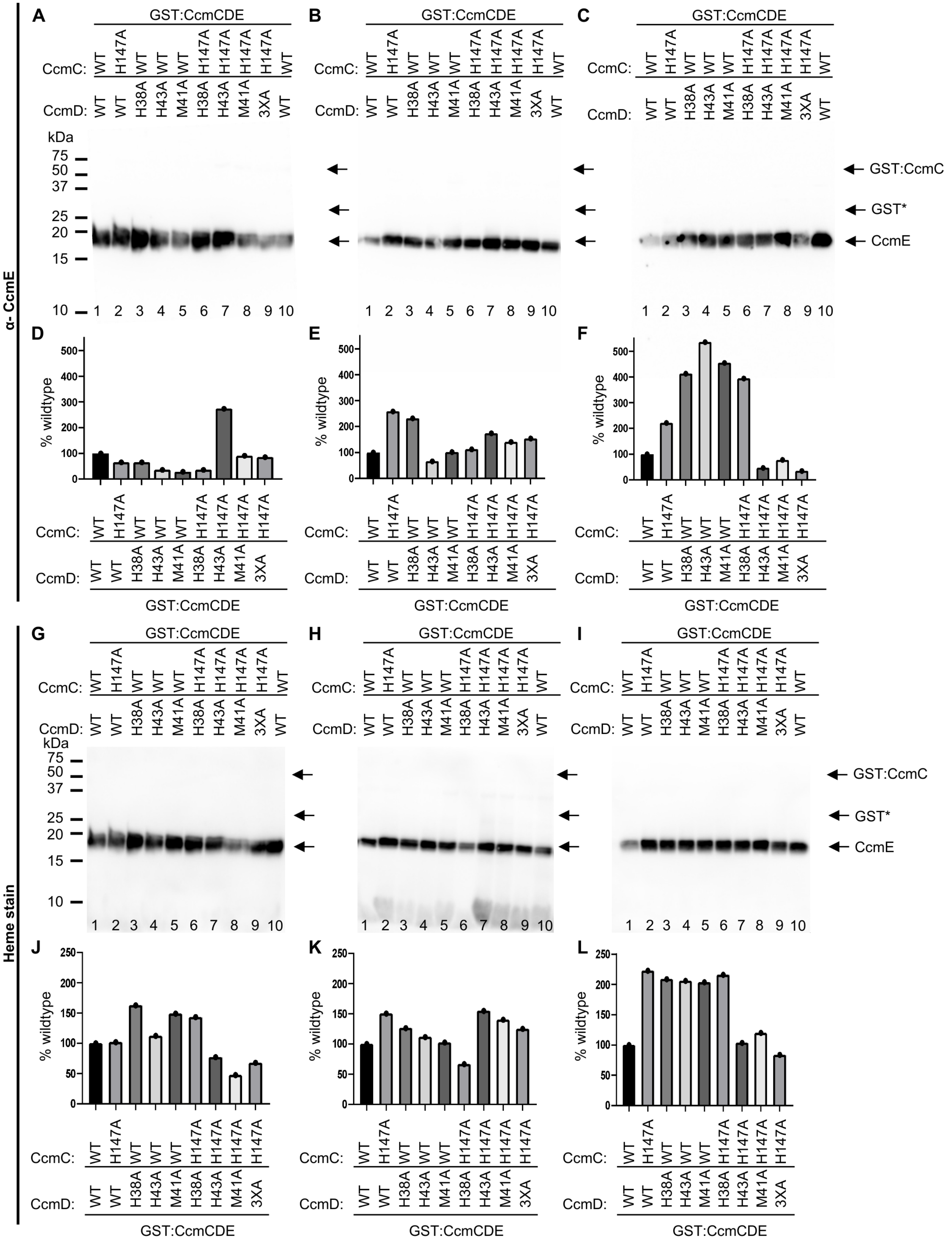

Supplemental Figure 2. Analysis of CcmE co-purification and holoCcmE formation by the CcmC/D alanine variants in the GST:CcmCDE genetic background. (A-L) Indicated single, double and triple alanine substitutions were engineered in CcmC and/or CcmD. 5 µg of affinity purified protein was separated via SDS-PAGE and assessed via (A-C) α-CcmE and (G-I) heme stain. (D-F) Quantification of CcmE co-purification via α-CcmE immunoblot. GST:CcmCDE wild type CcmE co-purification normalized to 100%. CcmE bands were quantified with AzureSpot (v2.2.167). (G-I) Quantification of formation of holoCcmE based on heme stain. As above, heme-stained band was quantified with AzureSpot. Each blot represents an independent biological replicate: (A, G) Replicate 1; (B, H) Replicate 2 ; (C, I) Replicate 3. Replicate 1 is also presented in Figure 2D, E.

Supplemental Figure 3

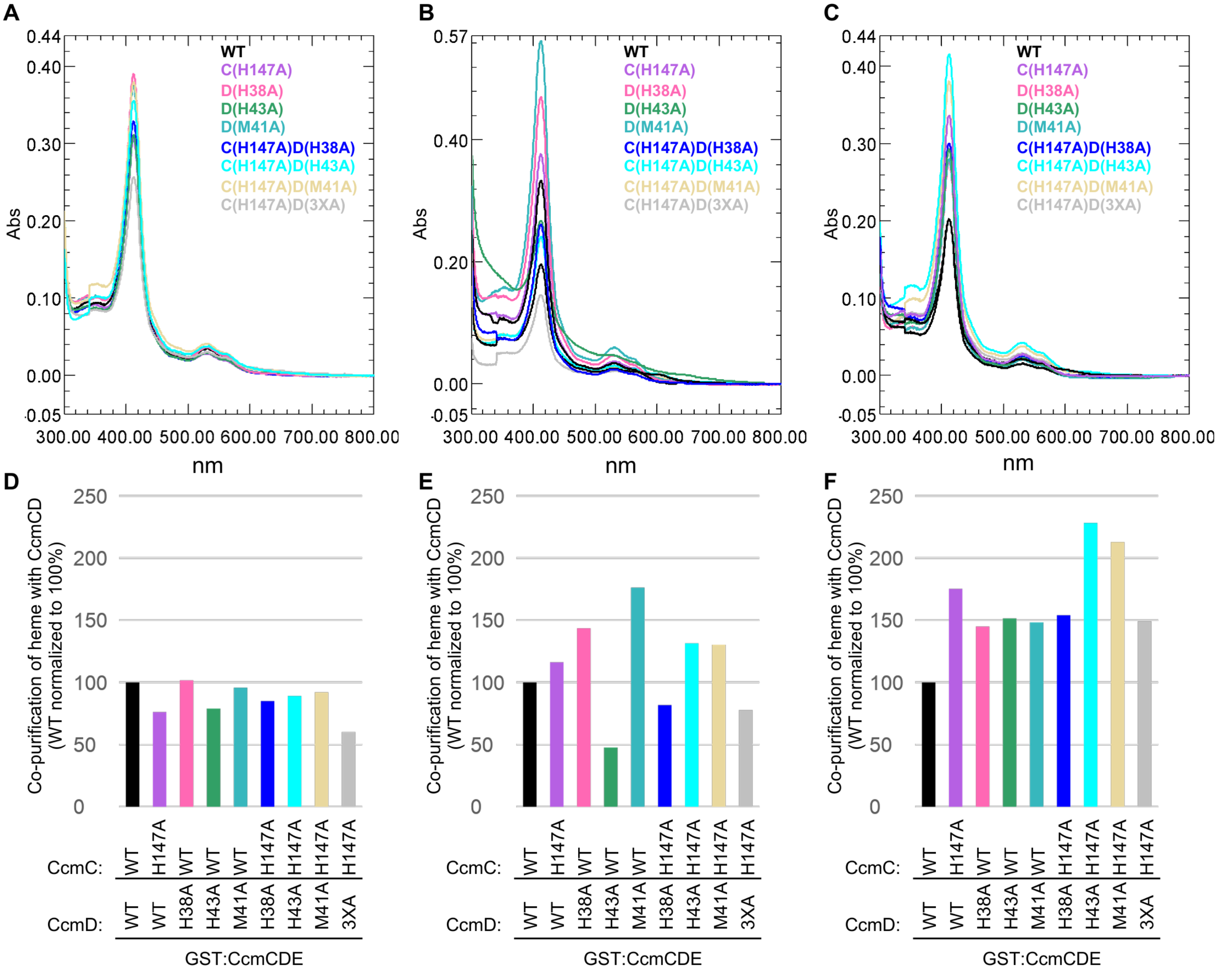

Supplemental Figure 3. UV-vis spectral analysis for heme co-purification by the CcmC/D alanine variants in the GST:CcmCDE genetic background. 50 µg of affinity purified protein was used to determine the relative heme copurification with GST:CcmCDE wildtype or alanine variants using the Soret peak height (412 nm) to valley (365 nm) measurements normalized to wildtype from as purified UV-vis spectra. (A-C) UV-vis spectra overlay of individual biological replicates: (A) Replicate 1; (B) Replicate 2; (C) Replicate 3. (D-F) Corresponding relative heme quantification measured from peak (412 nm) to valley (365 nm) and with wildtype normalized to 100%. Individual variants are color coded with the corresponding key: WT, C(H147A), D(H38A), D(H43A), D(M41A), C(H147A)D(H38A), C(H147A)D(H43A), C(H147A)D(M41A), C(H147A)D(3XA). Replicate 1 is also presented in Figure 2F.

Supplemental Figure 4

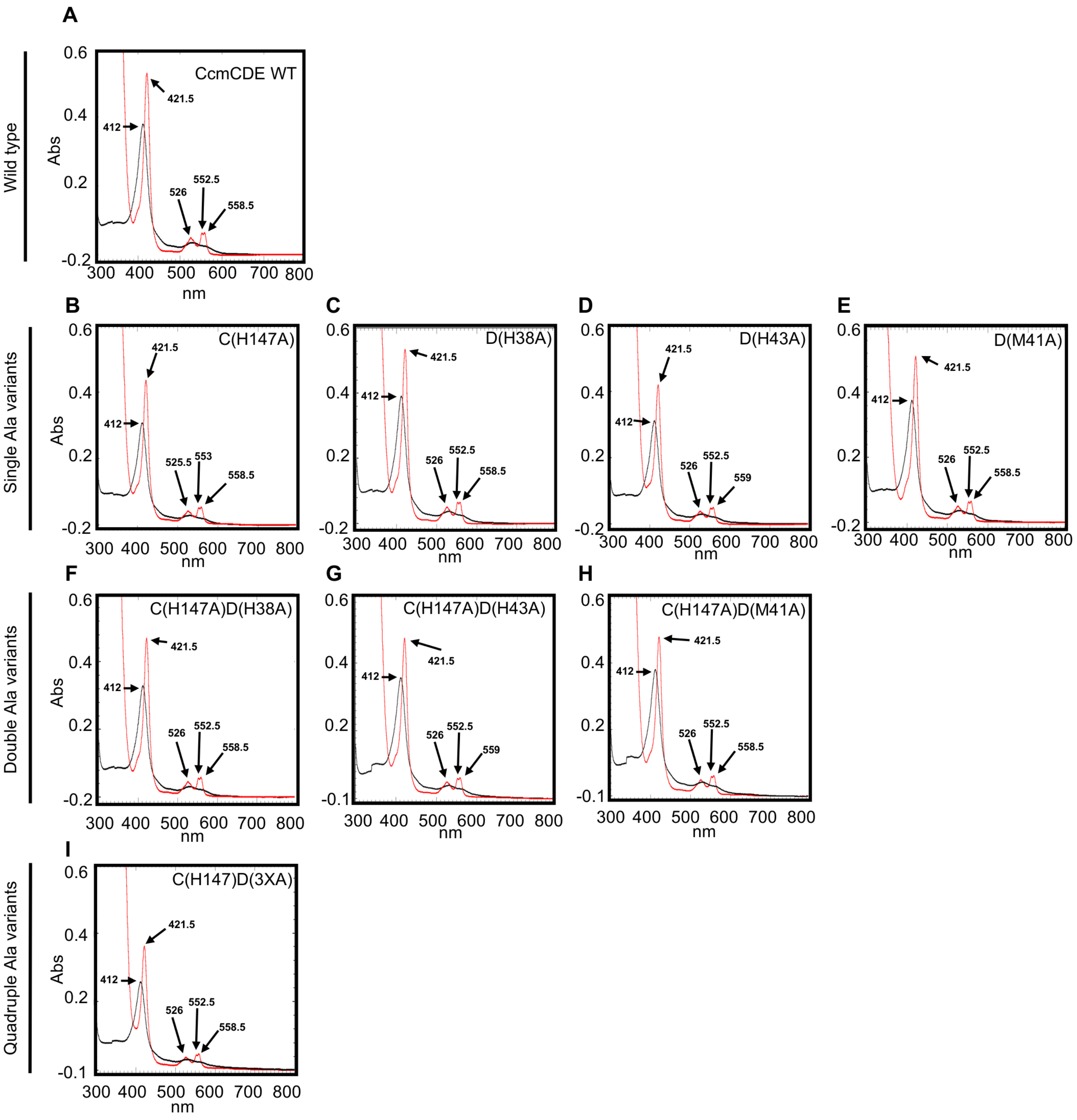

Supplemental Figure 4. CcmC and CcmD alanine variants do not disrupt the heme environment in GST:CcmCDE. (A-I) 50 μg of affinity purified protein was analyzed via scanning UV-vis spectral analysis from 800-300 nm. As purified spectra (black) and reduced spectra (red) are shown with key peaks indicated. Spilt alpha-peak (~553 and ~559) are characteristic of formation of holoCcmE (2).

Supplemental Figure 5

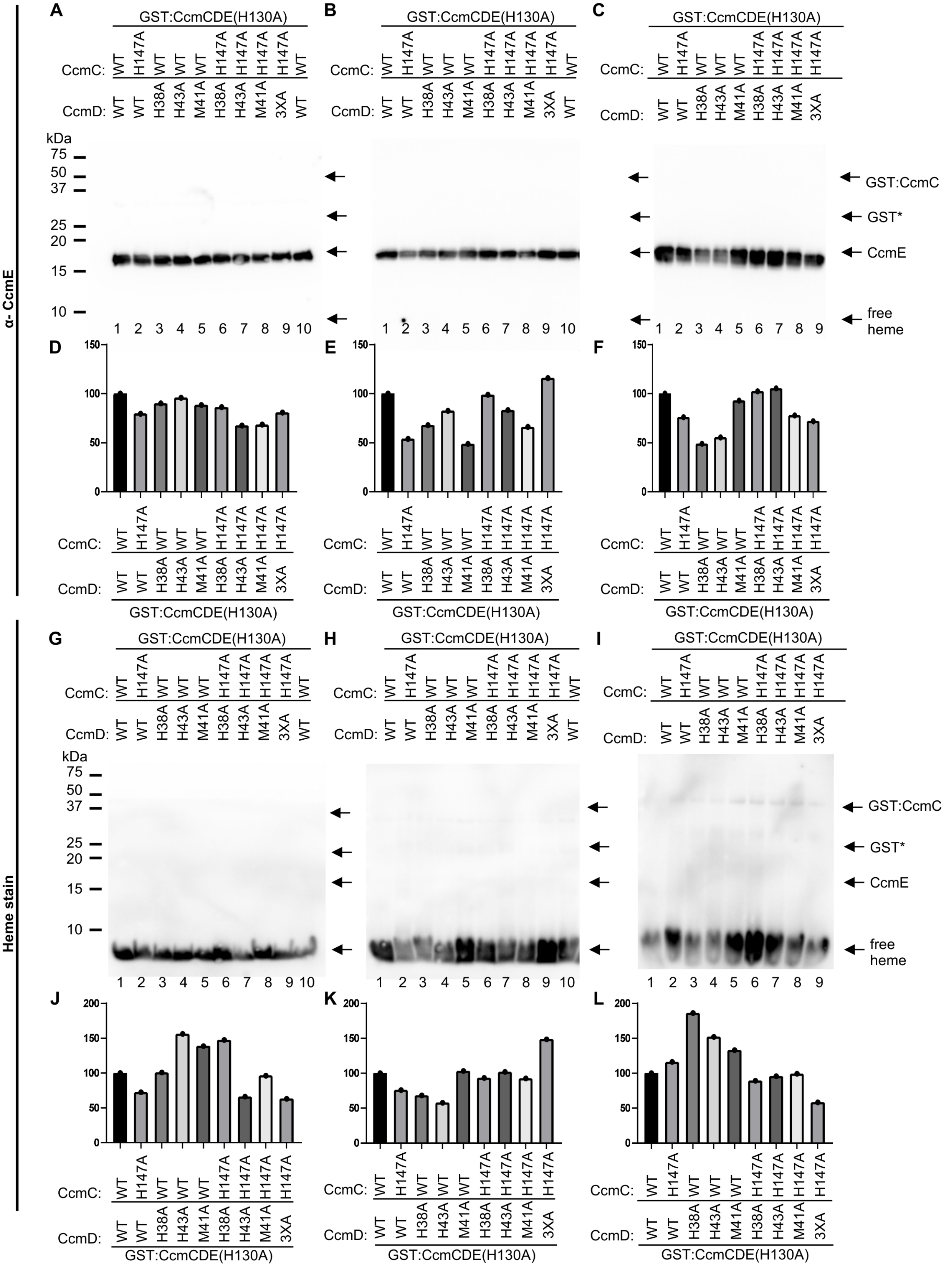

Supplemental Figure 5. Analysis of CcmE co-purification and heme co-purification by CcmC/D alanine variants in the GST:CcmCDE(H130A) genetic background. (A-L) Indicated single, double and triple alanine substitutions were engineered in CcmC and/or CcmD. 5 µg of affinity purified protein was separated via SDS-PAGE and assessed via (A-C) α-CcmE and (G-I) heme stain. (D-F) Quantification of CcmE co-purification via α-CcmE immunoblot. GST:CcmCDE wild type CcmE co-purification normalized to 100%. CcmE band was quantified with AzureSpot (v2.2.167). (G-I) Quantification of free heme via a heme stain. As above, heme-stained band was quantified with Azure spot. Each blot represents an individual biological replicate: (A, G) Replicate 1; (B, H) Replicate 2; (C, I) Replicate 3. Replicate 3 is also presented in Figure 3D, E.

Supplemental Figure 6

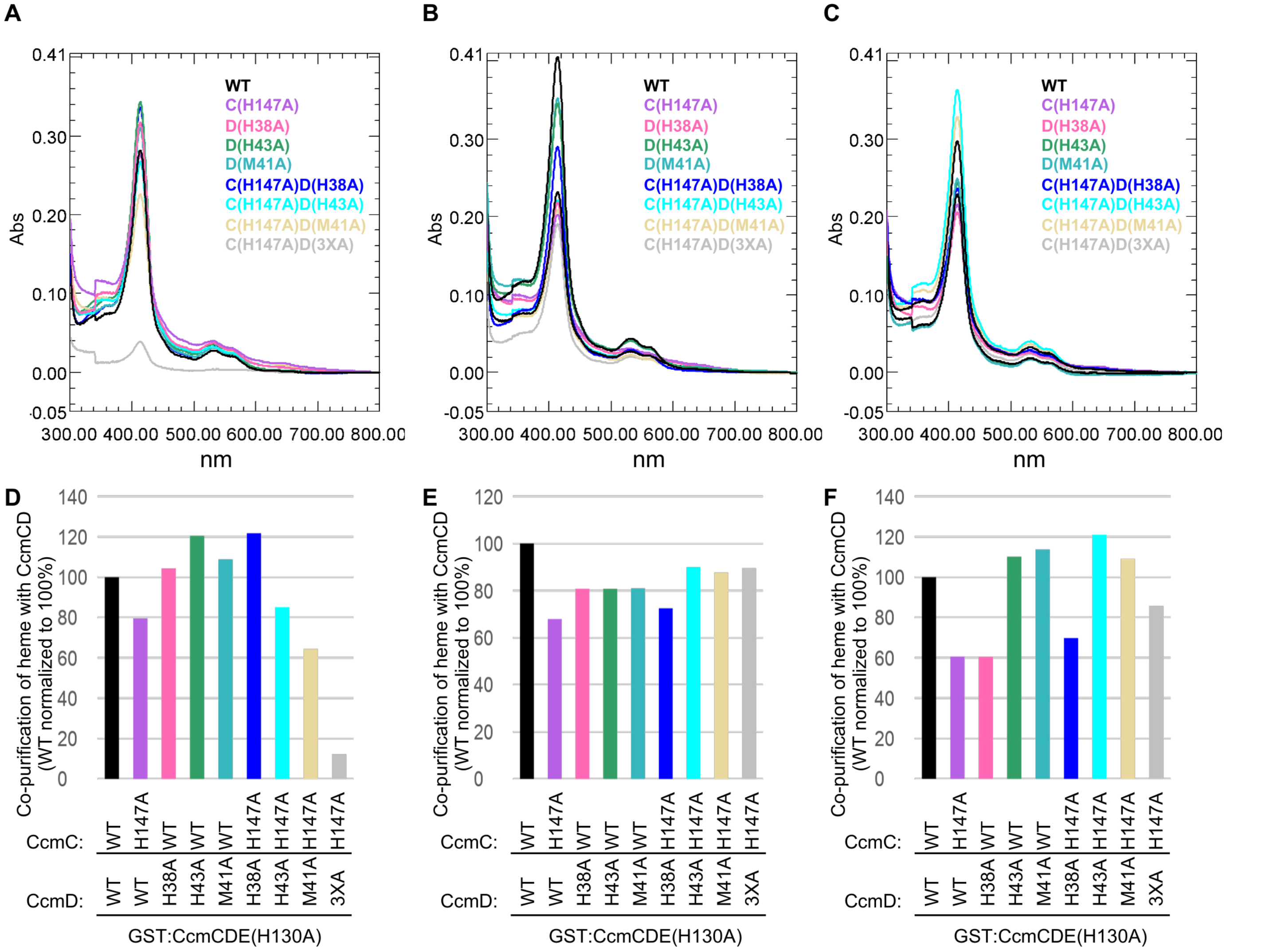

Supplemental Figure 6. UV-vis spectral analysis for heme co-purification with the CcmC/D alanine variants in the GST:CcmCDE(H130A) genetic background. 50 µg of affinity purified protein was used to determine the relative heme copurification with GST:CcmCDE(H130A) wildtype or alanine variants using the Soret peak height (412 nm) to valley (365 nm) measurements normalized to wildtype from as purified UV-vis spectra. (A-C) UV-vis spectra overlay of individual biological replicates: (A) Replicate 1; (B) Replicate 2; (C) Replicate 3. (D-F) Corresponding relative heme quantification measured from peak (412 nm) to valley (365 nm) and normalized to wildtype. Individual variants are color coded with the corresponding key: WT, C(H147A), D(H38A), D(H43A), D(M41A), C(H147A)D(H38A), C(H147A)D(H43A), C(H147A)D(M41A), C(H147A)D(3XA). Replicate 3 is also presented in Fig. 3F.

Supplemental Figure 7

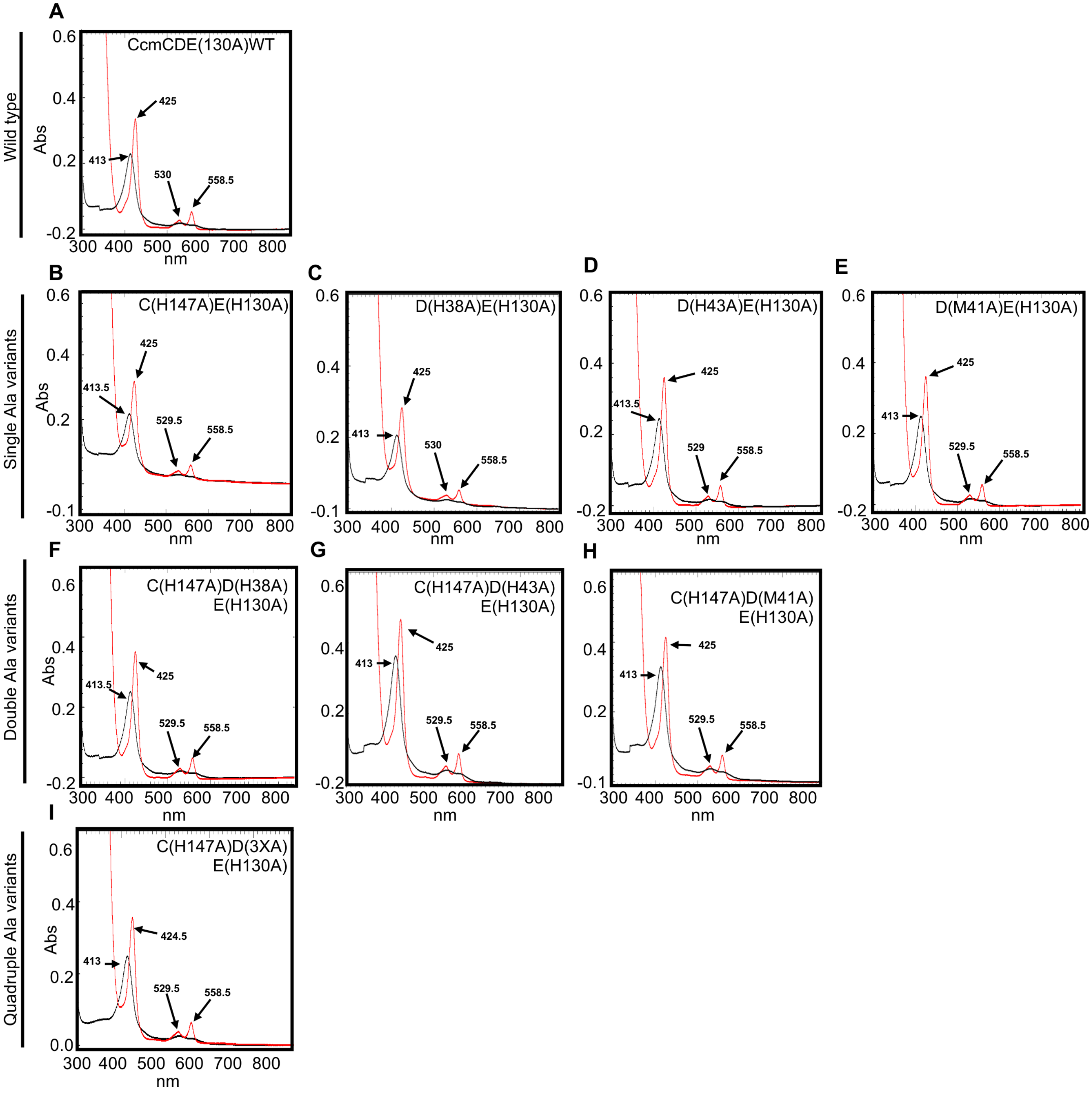

Supplemental Figure 7. CcmC and CcmD alanine variants do not disrupt the heme environment in GST:CcmCDE(H130A). (A-I) As described in Fig. S2, 50 µg of affinity purified protein was analyzed via scanning UV-vis spectral analysis from 800-300 nm. As purified spectra (black) and reduced spectra (red) are shown with key peaks indicated. CcmE H130A does not have a split alpha-peak.

Supplemental Table 1

Supplemental Table 1. Relevant Strains, Plasmids and Primers Employed in this Study

Strain, Plasmid	Description	Reference
E. coli		
RK103	*E. coli* MG1655 Δ*ccm::kan*^{R}, deleted for all *ccm* genes	*(3)*
NEB-5α	fhuA2 Δ(argF-lacZ)U169 phoA glnV44 Φ80 Δ(lacZ)M15 gyrA96 recA1 relA1 endA1 thi-1 hsdR17	
Plasmid		
pRGK332	pBAD cytochrome *c*4:His	*(3)*
pRGK375	pGEX GST:CcmCDE	*(2)*
pRGK380	pGST:GST:CcmCDE(H130A)	*(2)*
pMCS250	pGEX GST:CcmABCD(MBP:E)(F:His)GH	(1)
pMCS1096	pGEX GST:CcmC(H147A)DE	This Study
pMCS1098	pGEX GST:CcmCD(H38A)E	This Study
pMCS1100	pGEX GST:CcmC(H147A)D(H38A)E	This Study
pMCS1146	pGEX GST:CcmC(H147A)DE(H130A)	This Study
pMCS1148	pGEX GST:CcmCD(H38A)E(H130A)	This Study
pMCS1150	pGEX GST:CcmC(H147A)D(H38A)E(H130A)	This Study
pMCS1152	pGEX GST:CcmCD(H43A)E	This Study
pMCS1154	pGEX GST:CcmCD(M41)E	This Study
pMCS1156	pGEX GST:CcmC(H147A)D(H43A)E	This Study
pMCS1158	pGEX GST:CcmC(H147A)D(M41A)E	This Study
pMCS1160	pGEX GST:CcmCD(H43A)E(H130A)	This Study
pMCS1162	pGEX GST:CcmCD(M41A)E(H130A)	This Study
pMCS1164	pGEX GST:CcmC(H147A)D(H43A)E(H130A)	This Study
pMCS1166	pGEX GST:CcmC(H147A)D(M41A)E(H130A)	This Study
pMCS1198	pGEX GST:CcmABC(H147A)D(MBP:E)(F:His)GH	This Study
pMCS1199	pGEX GST:CcmABCD(H38A)(MBP:E)(F:His)GH	This Study
pMCS1200	pGEX GST:CcmABCD(H43A)(MBP:E)(F:His)GH	This Study
pMCS1201	pGEX GST:CcmABCD(M41A)(MBP:E)(F:His)GH	This Study
pMCS1203	pGEX GST:CcmABC(H147A)D(H43A)(MBP:E)(F:His)GH	This Study
pMCS1204	pGEX GST:CcmABC(H147A)D(M41A)(MBP:E)(F:His)GH	This Study
pMCS1443	pGEX GST:CcmABC(H147A)D(H38A)(MBP:E)(F:His)GH	This Study
pMCS1541	pGEX GST:CcmABC(H147A)D(H38A/M41A/H43A)(MBP:E)(F:His)GH	This Study
pMCS1547	pGEX GST:CcmC(H147A)D(H38A/M41A/H43A)E(H130A)	This Study
pMCS1551	pGEX GST:CcmC(H147A)D(H38A/M41A/H43A)E	This Study

Oligonucleotide	Sequence (5' --> 3')	Purpose (to clone)	Template
MSP700	cggtcgtcgaaggcggcccacagggcaatcacaccc	pMCS1096	pRGK375
		pMCS1100	pMCS1098
		pMCS1146	pRGK380
		pMCS1156	pMCS1152
		pMCS1158	pMCS1154

		pMCS1164	pMCS1160
		pMCS1166	pMCS1162
		pMCS1198	pMCS250
		pMCS1204	pMCS1201
		pMCS1443	pMCS1199
MSP701	gggtgtgattgccctgtgggccgccttcgacgaccg	pMCS1096	pRGK375
		pMCS1100	pMCS1098
		pMCS1146	pRGK380
		pMCS1156	pMCS1152
		pMCS1158	pMCS1154
		pMCS1164	pMCS1160
		pMCS1166	pMCS1162
		pMCS1198	pMCS250
		pMCS1204	pMCS1201
		pMCS1443	pMCS1199
MSP706	gcgcgatgttgcatcaccgaggccacgaccaaaaccacc	pMCS1098	pRGK375
		pMCS1199	pMCS250
MSP707	ggtggttttggtcgtggcctcggtgatgcaacatcgcgc	pMCS1098	pRGK375
		pMCS1199	pMCS250
MSP750	gaattgcgcgatgttgcgccaccgagtgcacgacc	pMCS1154	pRGK375
		pMCS1162	pRGK380
		pMCS1201	pMCS250
MSP751	ggtcgtgcactcggtggcgcaacatcgcgcaattc	pMCS1154	pRGK375
		pMCS1162	pRGK380
		pMCS1201	pMCS250
MSP746	cagaattgcgcgagcttgcatcaccgagtgcacgac	pMCS1152	pRGK375
		pMCS1160	pRGK380
		pMCS1200	pMCS250
		pMCS1203	pMCS1198
MSP747	gtcgtgcactcggtgatgcaagctcgcgcaattctg	pMCS1152	pRGK375
		pMCS1160	pRGK380
		pMCS1200	pMCS250
		pMCS1203	pMCS1198
MSP744	ggcgtatagttttcatcggctttcgccagcacttctttcgc	pMCS1148	pMCS1098
		pMCS1150	pMCS1100
MSP745	gcgaaagaagtgctggcgaaagccgatgaaaactatacgcc	pMCS1148	pMCS1098
		pMCS1150	pMCS1100
MSP953	ccgctggtggttttggtcgtggcctcggtggcgcaagctcgcgcaattctgcg	pMCS1551	pMCS1158
		pMCS1547	pMCS1166
		pMCS1541	pMCS1024
MSP954	cgcagaattgcgcgagcttgcgccaccgaggccacgaccaaaaccaccagcgg	pMCS1551	pMCS1158
		pMCS1547	pMCS1166
		pMCS1541	pMCS1024

Supplemental References

1. Kreiman AN, Garner SE, Carroll SC, Sutherland MC. 2025. Biochemical mapping reveals a conserved heme transport mechanism via CcmCD in System I bacterial cytochrome *c* biogenesis. mBio 16:e03515-24.

2. Richard-Fogal CL, Frawley ER, Bonner ER, Zhu H, San Francisco B, Kranz RG. 2009. A conserved haem redox and trafficking pathway for cofactor attachment. EMBO J 28:2349–2359.

3. Feissner RE, Richard-Fogal CL, Frawley ER, Loughman JA, Earley KW, Kranz RG. 2006. Recombinant cytochromes c biogenesis systems I and II and analysis of haem delivery pathways in Escherichia coli. Mol Microbiol 60:563–577.
